# Outcomes of severe acute kidney injury requiring renal replacement therapy in renal transplant patients: A single center experience

**DOI:** 10.12669/pjms.41.3.10371

**Published:** 2025-03

**Authors:** Abdul Rauf Hafeez, Ranjeet Kumar, Nazarul Hassan Jafry, Muniba Rehman

**Affiliations:** 1 Abdul Rauf Hafeez Associate Professor, Sindh Institute of Urology and Transplantation (SIUT), Karachi, Pakistan; 2 Ranjeet Kumar, Assistant Professor, Sindh Institute of Urology and Transplantation (SIUT), Karachi, Pakistan; 3 Nazarul Hassan Jafry Professor, Sindh Institute of Urology and Transplantation (SIUT), Karachi, Pakistan; 4 Muniba Rehman Lecturer, Sindh Institute of Urology and Transplantation (SIUT), Karachi, Pakistan

**Keywords:** Renal transplant, Severe acute kidney injury, Outcome

## Abstract

**Objective:**

We aimed to assess the renal allograft and patient survival following acute kidney injury requiring dialysis therapy.

**Methods:**

We analyzed the medical record of 3000 first living donor kidney transplant performed between 2008 to 2017 for AKI requiring dialysis at Sindh Institute of Urology and Transplantation, Karachi, Pakistan. Patients less than 15 years of age and those AKI events that happened less than three months post renal transplant were excluded. Renal allograft and patient survival were recorded at discharge and one-year post AKI. Recovery of renal functions was assessed at three-month.

**Results:**

AKI requiring dialysis therapy was identified in 154 (5.1%) patients. At discharge, 115 (74.7%) were alive and 71 (61.7%) of them were dialysis free. At three-month, out of 71 dialysis free patients, 11 (15.5%) had complete recovery, 54 (76%) had partial recovery and six (8.5%) required dialysis again. At one-year, 98 (63.6%) patients were alive and 42 (42.9%) of them were dialysis free. Infectious etiology of AKI (*P*= 0.000; 0R 6.00; CI, 2.3-15.08) and more than two non-dialysis -requiring AKI in the past (*P=* 0.017; OR 3.04; CI, 1.2-7.5) were the risk factors of in-hospital mortality. Non-infectious cause of AKI (*P*=0.000; OR 45.5; CI, 9.9-206) and being off calcineurin inhibitors (*P*=0.014; OR 4.4; CI, 1.3-14.8) were the risk factors of dialysis dependency at hospital discharge.

**Conclusions:**

Dialysis-requiring AKI secondary to infectious etiology has both high mortality and chances of recovery in survivors. They need prompt diagnosis and treatment. Non-infectious etiology and being off CNI are the risk factors of graft loss in dialysis-requiring AKI.

## INTRODUCTION

In the non-transplant setting, the incidence of acute kidney injury (AKI) is about 4-20% in hospitalized patients and there is a close association between AKI severity and prolonged hospitalization, end stage renal disease (ESRD) and death.^1^-^3^ Renal transplantation is the best therapeutic option for end stage renal failure (ESRF) patients^4^ but renal grafts are more prone to AKI, including more severe forms and dialysis-requiring AKI. In addition to transplant specific insults like ischemic-reperfusion injury, drug toxicities, immunological and surgical complications, all forms of AKI seen in native kidneys can affect renal grafts.^5^

The incidence of AKI in renal transplants has been reported from 11% to 82.3%^5^ and it is independently associated with increased graft loss from any cause and death.^6^ Most of the studies addressing this issue are from the West where health facilities are adequate, generally included patients with milder form of AKI and less representation of dialysis-requiring AKI. So, we decided to perform this study to assess the renal allograft and patient survival following dialysis-requiring AKI at hospital discharge and one-year, in the setting of low income country.

## METHODS

We performed a retrospective analysis of medical records of 3000 (79.5% male) living donor renal transplants performed between 2008 to 2017 for AKI admission requiring dialysis therapy at Sindh Institute of Urology and Transplantation, Karachi, Pakistan. We started the analysis three months after kidney transplant when renal function was usually stabilized. Patients less than 15 years of age were excluded. Donor, recipient and transplant variables (Table-I) along with the cause of dialysis-requiring AKI were recorded and patients were followed up to 12-months post AKI event.

**Table-I:** Recipient, donor and transplant characteristics.

Variables	Dialysis-Requiring AKI (n=154)
***Recipient Age at transplant (*n,* %)***	
15-30 years	90 (58.4)
> 30 years	64 (41.6)
Recipient gender (male %)	135 (87.7)
***Cause of ESRD (*n*, %)***	
Uncertain cause	118 (76.6)
Diabetes mellitus	3 (1.9)
Chronic glomerulonephritis	13 (8.4)
Stone disease	18 (11.7)
Adult polycystic kidney disease	1 (0.6)
Obstetrical complication	1 (0.6)
***HLA mismatches (*n*, %)***	
≤ 3	132 985.7)
> 3	22 (14.3)
***Index hospital days (*n,* %)***	
≤ 12 days	93 (60.4)
> 12 days	61 (39.6)
***Discharged creatinine (*n,* %)***	
≤ 1.2 mg/dl	82 (53.2)
>1.2 mg/dl	72 (46.8)
***Biopsy proven rejection (*n,* %)***	
Absent	82 (53.2)
Present	72 (46.8)
***Non dialysis requiring AKI (*n,* %)***	
≤ 2 episodes	62 (40.3)
> 2 episodes	92 (59.7)
***Duration of transplant (*n,* %)***	
< 1 year	9 (5.8)
1-3 years	45 (29.2)
3-6 years	100 (64.9)
***Calcineurin inhibiotor use (*n,* %)***	
Cyclosporine	59 (38.3)
Tacrolimus	66 (42.9)
None	29 (18.8)
Mean baseline serum creatinine (mg/dl)	1.86 (±0.69)
Mean baseline estimated GFR (ml/min/1.73 m^2^)	56.29 (± 25.12)
***Mean Baseline estimated GFR (*n,* %)***	
≥60	60 (39)
< 60	94 (61)
***Donor age (n, %)***	
≤ 30 years	57 (37)
> 30 years	97 (63)
Donor gender (male %)	78 (50.6)
***Donor GFR (n, %)***	
≥ 100 ml/min/1.73m^2^	100 (64.9)
<100 ml/min/1.73m^2^	54 (35.1)

GFR: glomerular filtration rate, ESRD: end stage renal disease, HLA: human leukocyte antigen.

### Ethical Approval:

The study was approved by local ethical committee (SIUT-ERC-November, 29 2021/A-349).

Baseline serum creatinine was calculated by taking mean of three stable creatinine levels checked in outpatient clinic at least one month apart before the AKI event. Baseline GFR was calculated by CKD-EPI (Creatinine-based, 2021) equation**.** Non-dialysis-requiring AKI in the past are all those hospital admissions that were associated with acute rise in serum creatinine level from baseline at admission or during admission from any cause but didn’t require hemodialysis therapy. Causes of dialysis-requiring AKI were classified as infectious and non-infectious. Non-infectious causes were further divided as rejection and others.

The survival of patient and dialysis independence were assessed at hospital discharge and one-year post AKI event. Those dialysis independent patients who remained alive at three months, their GFR was estimated by CKD-EPI (Cr based 2021) equation and compared with baseline estimated GFR to classify as complete and partial recovery. Complete renal recovery was defined as the gain of lost kidney function to pre-AKI baseline levels of estimated GFR (±10%). Recovery of renal function was defined as partial if a persistent change of GFR from baseline remained evident, but did not require renal replacement therapy.

### Statistical analysis:

Data was analyzed using IBM SPSS statistics software (version 22.0) (SPSS Inc., Chicago, IL, USA). Continuous variables were reported as mean ± standard deviation (SD) whereas categorical variables were reported as frequencies and percentages. The analysis of factors associated with in-hospital mortality and dialysis dependency at discharge was performed by using Pearson’s Chi-square test or Fishers test. Variables with *P* values ≤ 0.10 were included in multiple logistic regression models and variables with *P* values < 0.05 were considered statistically significant.

## RESULTS

Admissions with AKI requiring dialysis were identified in 154 (5.1%) transplant recipients. Out of 154 recipients, 135 (87.7%) were male. The mean age of recipient was 30.51±9.33 years. The mean baseline estimated GFR was 56.29 (SD 25.12) ml/min/1.73 m^2^. Out of 154 recipients, 29 (18.8%) recipients were off calcineurin inhibitor (CNI) and 92 (59.7 %) had more than two episodes of non-dialysis-requiring AKI in the past (Table-I).

**Fig.1:**
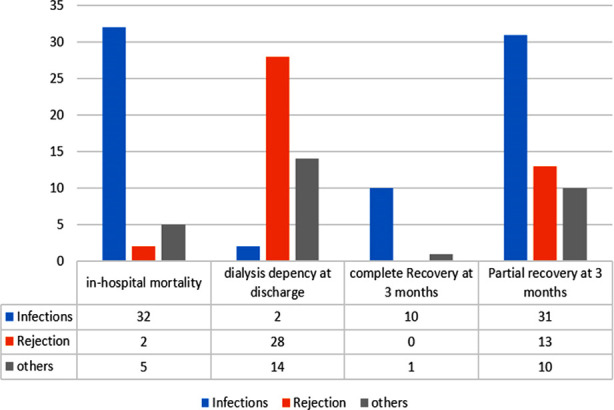
In-hospital mortality, dialysis dependency at discharge and recovery pattern at three-month post AKI in different etiological group.

Urinary tract infection was the most common etiology of AKI requiring dialysis (30.5%) followed by plasma cell rich acute cellular rejection (22.7%) (Table-II). At discharge, 115 (74.7%) patients were alive and 39 (25.3%) had in-hospital mortality. Among survivors, 71 (61.7%) were dialysis free. Majority of the expired patients had infectious etiology and large number of surviving but dialysis dependent patients had noninfectious etiology of AKI (Fig.1). At three-month post AKI, out of 71 dialysis free patients, 11 (15.5%) had complete recovery, 54 (76%) had partial recovery and six (8.5%) required dialysis again. Majority of the patients with complete and partial recovery had infectious etiology of AKI (Fig.1). At one-year post AKI event, 98 (63.6%) patients were alive and 42 (42.9%) of them were dialysis free.

**Table-II:** Causes of dialysis-requiring AKI.

Etiology	Frequency	Percentage
**Infections**	79	51.3
1.Urinary Tract Infection	47	30.5
2.Respiratory Tract Infection	19	12.3
3.CNS Infection	2	1.3
4.Skin Abscess	7	4.5
5.Acute Gastroenteritis	8	5.2
**Rejections**	45	29.2
1.Acute Vascular Rejection	4	2.6
2.Plasma Cells Rich Acute Cellular Rejection	35	22.7
**Others**	30	19.2
1.Hemolytic Uremic Syndrome	9	5.8
2.Focal Segmental Glomerulosclerosis (FSGS)	11	7.1
3.Glomerulonephritis other than FSGS	3	1.9
4.Obstructive Nephropathy	4	2.6
5.Miscellaneous	5	3.2

In univariate analysis, we identified following variables associated with in-hospital mortality: Infectious etiology of AKI requiring dialysis, more than two episodes of non-dialysis-requiring AKI in the past, index hospitalization of more than 12 days, recipient age more than 30 years and duration of transplant more than three years. In multivariate analysis of risk factors, Infectious etiology of AKI (*P*= 0.00; 0R 6.00; CI, 2.3-15.08) and more than two episodes of non-dialysis-requiring AKI in the past (*P=* 0.01; OR 3.04; CI, 1.2-7.5) were the two independent risk factors of in-hospital mortality (Table-III).

**Table-III:** Univariate and multivariate analysis of risk factors of in-hospital mortality.

Risk factors	Non Survivors %	Survivors %	Unadjusted OR (95% CI), P value	Adjusted OR (95% CI), P value
Infectious etiology of AKI	82.1	40.9	2.1 (1.5-2.7) 0.00	6.0 (2.3-15), 0.00
Non-dialysis-requiring AKI in the past (> 2 times)	79.5	53	1.4(1.1-1.8), 0.004	3.0 (1.2-7.5), 0.01
Index hospital days (>12 days)	51.3	35.7	1.4 (0.9-2.1), 0.06	1.1(0.3-1.5), 0.48
Recipient Age (> 30 years)	51.3	38.3	1.3 (0.9-1.9), 0.10	1.2(0.5-2.8), 0.61
Duration of transplant (> 3 years)	10.3	21.7	0.4 (0.1-1.2), 0.08	0.5 (0.2-1.2),0.14
Recipient gender (male)	84.6	88.7	0.9 (0.8-1.1), 0.3	
Dialysis duration (> 6months)	25.6	32.2	0.7 (0.4-1.4), 0.29	
Donor age (> 30 years)	61.5	63.5	0.9 (0.7-1.2), 0.48	
Donor gender (male)	51.3	50.4	1.0 (0.7-1.4), 0.53	
Donor GFR (≤ 100 ml/min)	35.9	34.8	1.0 (0.6-1.6), 0.52	
HLA mismatches (> 3)	20.5	12.2	1.6 (0.7-3.7), 0.15	
Discharged creatinine (> 1.2 mg/dl)	46.2	47	0.9 (0.6-1.4), 0.54	
Biopsy proven rejection (yes)	48.7	46.1	1.0 (0.7-1.5), 0.46	
Off Calcineurin inhibitor	71.8	61.9	1.2(0.9-1.5), 0.15	
Mean Baseline GFR	66.7	59.1	1.1 (0.8-1.4), 0.26	

We found following variables associated with dialysis dependency at discharge in univariate analysis: Non-infectious cause of dialysis-requiring AKI, being off CNI, recipient age ≤ 30 years and ≤ two episodes of non-dialysis-requiring AKI in the past. Non-infectious cause of AKI (*P*=0.000; OR 45.5; CI, 9.9-206) and being off CNI (*P*=0.014; OR 4.4; CI, 1.3-14.8) were the independent risk factors of dialysis dependency at discharge (Table-IV).

**Table-IV:** Univariate and multivariate analysis of risk factors of dialysis dependency at discharge.

Risk factors	Dialysis dependency at discharge %	Dialysis free at discharge %	Unadjusted OR (95% CI), P value	Adjusted OR (95% CI), P value
Non-Infectious etiology of AKI	97.7	30.9	3.1 (2.3-4.1), 0.000	45(9.9-206), 0.000
Off Calcineurin inhibitor	34.1	12.7	2.6 (1.4-5.0), 0.003	4.4 (1.3-14.8), 0.014
Recipient Age (≤ 30 years)	70.5	53.6	1.3 (1.0-1.7), 0.04	1.3(0.4-3.9),0.56
Non-dialysis-requiring AKI in the past (≤ 2 times)	54.5	34.5	1.5 (1.0-2.2), 0.018	1.9 (0.7-5.1), 0.17
Mean Baseline GFR (≤ 60 ml/min)	59.1	61.8	0.9 (0.7-1.2), 0.446	
Duration of transplant (> 3 years)	61.4	64.5	0.9 (0.7-1.2), 0.42	
Recipient gender (male)	93.2	85.5	1.1 (0.9-1.2), 0.14	
Donor age (> 30 years)	61.4	63.6	0.9 (0.7-1.2), 0.46	
Donor gender (male)%	54.4	49.1	1.2 (0.7-1.5), 0.33	
Donor GFR (≤ 100 ml/min)	31.8	36.4	0.8 (0.5-1.4), 0.36	
HLA mismatches (> 3)	11.4	15.5	0.7 (0.2-1.8), 0.35	
Index hospital days (>12 days)	36.4	40.9	0.8 (0.5-1.3), 0.36	
Discharged creatinine (> 1.2 mg/dl)	45.5	47.3	0.9 (0.6-1.4), 0.49	
Biopsy proven rejection (yes)	47	46.4	1.0(0.7-1.4), 0.51	

## DISCUSSION

AKI in renal transplant is a common problem. In this study, the incidence of dialysis-requiring AKI was 5.1% and UTI was the most common etiology. A low incidence i.e. 1.66% of dialysis-requiring AKI was reported earlier in 27,232 patients in first three-years post-transplant.^6^ As AKI events were recorded in first three years only, it could be the reason of low incidence in this study comparing to our results. UTI was one of the risk factors of severe AKI^7^ and the most common cause of AKI in another study^8^ which is consistent with our finding. Idrees et al^9^ from Pakistan found that biopsy proven renal graft pyelonephritis is associated with partial or complete loss of graft functions and another study from Pakistan reported that UTI incidence in renal transplant was 26.7% and associated with loss of graft functions at one and five years post event.^10^ Our measured In-hospital mortality was 25.3% and one-year mortality was 34.4% which is not higher than the reported mortality in transplant literature. A Swiss study which had renal and non-renal solid organ transplant patients concluded that 90-day mortality was 49% following AKI requiring dialysis.^11^ A Brazilian study assessed the mortality predictors in 190 septic renal transplant patients, out of which 40.5% required dialysis. It had 38.4% in-hospital and 42.6% one-year mortality.^12^

In the present study, 98 (63.6%) patients were alive at one-year and 56 (57.1%) of them were on dialysis therapy. A Colombian study^7^ documented that 12 (40%) patients had transplant loss at one-year out of 30 AKI stage three patients. A Japanese study^8^ that assessed renal graft survival following AKI of all intensities found that 21 (36%) had graft loss at 10-month interval and those patients who had severe AKI lost more grafts. As all our patients had severe AKI since start so we can expect more graft loss in our setting.

Traditional transplant variables i.e. previous rejection, immunosuppressive regime and Cytomegalovirus disease were described having no prognostic value for AKI mortality.^12^ The same was observed in our study in which past rejection episodes, immunosuppressive regime had no impact on mortality. Nakamura et al^8^ noted that donor age and relationship with recipient didn’t have association with transplant loss following AKI. We observed that donor age, gender, GFR and HLA mismatches didn’t influence patient and graft survival following severe AKI. No correlation was established between baseline estimated GFR and mortality in our study. The same was reported by Mehrotra et al^6^ in 453 renal transplant patients with AKI requiring dialysis.

The association between baseline GFR and transplant loss following AKI has been reported differently. Mehrota et al^6^ found that transplant loss from any cause was highest in higher levels of GFR regardless of AKI severity. Lower baseline GFR was one of the risk factors of dialysis dependency at hospital discharge in renal transplant patients in Filiponi et al^13^ study. Nakamura et al^8^ who studied graft failure following AKI in 289 renal transplants with living donors found that lower baseline GFR is associated with graft loss but this association is lost in severe form of AKI. Our results agreed with this finding as baseline GFR had no prognostic value for transplant loss in our study population.

Prior AKI insult that didn’t require dialysis and infections as admission cause were the two independent predictors of in-hospital mortality in our study. Wald et al^14^ who assessed the risk of chronic dialysis and death following non-dialysis-requiring AKI found that the absolute risk of death in survivors was more than eight times the rate of chronic dialysis. Secondly, infectious complications increased the mortality considerably in renal transplant patients when compared with non-infectious counterpart (62.9% vs 26.5%).^15^ Sepsis is one of the risk factors for death identified by Charboney et al^11^ in solid organ transplanted patients which is in alignment with our results. The acute rejection was an independent predictor of dialysis dependency at hospital discharge following AKI in renal transplant patients in Filiponi et al and Nakamura et al studies.^13^,^8^ In our analysis, acute rejection is also a risk factor for dialysis dependency at hospital discharge. Tacrolimus (calcineurin inhibiotors) withdrawal is associated with acute rejection episode and the formation of anti-HLA antibodies.^16^ Our patients who were at low risk of rejection and their CNI were withdrawal to avoid long term CNI toxicity had more graft loss when developed AKI**.**

### Limitation:

This is the first study from Pakistan that exclusively includes those renal transplant patients that developed AKI and required dialysis in which patient and graft survival was recorded. Additionally, we have documented the recovery trend at three months in different etiological groups that’s not reported earlier. But it has some limitations too. First, it was a retrospective, single centered study. Secondly, we couldn’t report the risk factors of dialysis-requiring AKI. Regarding the etiology, UTI and plasma cell rich acute cellular rejection which are the main causes of dialysis-requiring AKI in this study need further exploration for better understanding.

## CONCLUSION

Dialysis-requiring AKI secondary to infectious etiology has both high mortality and chances of recovery in survivors. They need prompt diagnosis and treatment. Noninfectious causes and being off CNI are the risk factors of graft loss in dialysis-requiring AKI in transplant setting.

### Authors contribution:

**AR:** Conceived and designed the study, involved in data collection, performed statistical analysis and writing the manuscript.

**RK, NJ and MR:** Collected the data, critical reiew and preparation of manuscript.

All authors have read, approved the final manuscript and are responsible for the integrity of the study.
